# Successful treatment of pediatric osteochondritis dissecans of the knee using a wrist arthroscope: A case report with long term follow-up

**DOI:** 10.1016/j.ijscr.2025.111196

**Published:** 2025-03-24

**Authors:** Stefanus Hengkie Marseno, Tri Wahyu Martanto, Hizbillah Yazid, Arif Zulkarnain, Kukuh Dwiputra Hernugrahanto, Julian Benedict Swannjo

**Affiliations:** a Department of Orthopedics and Traumatology, Faculty of Medicine, Universitas Airlangga, Surabaya, Indonesia; b Department of Orthopedics and Traumatology, Dr. Soetomo General Academic Hospital, Surabaya, Indonesia; c Faculty of Medicine, Universitas Airlangga, Surabaya, Indonesia

**Keywords:** Osteochondritis dissecans, Pediatric knee arthroscopy, Wrist arthroscope, Good health and well-being, Case report

## Abstract

**Introduction and importance:**

Osteochondritis dissecans (OCD) is a rare joint disorder that affects the subchondral bone and surrounding articular cartilage, resulting in localized necrosis and possible detachment of both bone and cartilage pieces. It primarily manifests in the knee joints of juvenile and teenage patients, and is frequently associated with recurring joint stress.

**Case presentation:**

An 8-year-old child presented with a three-month history of left knee pain, exacerbated by movement, which resulted in a limp. Clinical examination revealed knee edema, limited range of motion, and 30-degree extension limitation. Imaging revealed a chondral defect in the medial condyle accompanied by a detached cartilage flap. Arthroscopic surgery was performed using a wrist arthroscope for debridement and microfractures, followed by fibrin glue application. Postoperatively, the patient showed considerable improvement, achieving complete restoration of knee mobility, and a Lysholm knee score of 93.

**Clinical discussion:**

Addressing OCD in pediatric patients presents distinct problems owing to their reduced joint size and open growth plates. Wrist arthroscopes enhance precision in pediatric patients and reduce harm to adjacent cartilage and soft tissues. Conservative therapy may be sufficient for stable lesions; however, surgical intervention is required in unstable cases.

**Conclusion:**

This case illustrates the effective use of wrist arthroscope for the management of pediatric knee diseases. The small equipment facilitated accurate and efficient intervention, improved outcomes, and emphasized the importance of properly sized tools in pediatric orthopedic treatments.

## Highlights

• Osteochondritis Dissecans (OCD) is a rare pediatric joint disorder primarily affecting the knee, particularly the medial femoral condyle, and is characterized by localized bone and cartilage necrosis. It predominantly occurs in males (2:1 ratio) and is often linked to repetitive joint stress from sports activities.
• The use of a wrist arthroscope in pediatric OCD treatment allowed for precise debridement and microfracture of the chondral defect. This smaller, pediatric-friendly tool minimized damage to surrounding tissues, addressing challenges posed by the smaller joint size and open growth plates in children.
• The patient achieved significant post-operative improvement, with full range of motion restored within two months, a Lysholm knee score of 93, and a Tegner activity score of 4. This underscores the effectiveness of tailored surgical tools in optimizing outcomes for pediatric knee conditions.
• This case highlights the importance of developing specialized instruments for pediatric orthopedic procedures to improve precision, minimize complications, and ensure better functional outcomes in young patients.

## 1 Introduction and importance

Osteochondritis dissecans is a joint disease that affects the subchondral bone and the surrounding articular cartilage. This condition is caused by localized sections of bone necrosis, ultimately leading to the loosening of fragments of bone and cartilage into the joint. “Osteochondritis” refers to the inflammation of bone and cartilage. “Dissecans” refers to separation. In OCD, portions of the bone and cartilage may come loose, leading to pain, swelling, and reduced joint mobility [1].

It is a fairly rare disease, with an estimated 15–30 cases per 100,000 individuals. OCD primarily affects pediatric and adolescent patients. Most patients ranged in age from 10 to 20 years. OCD typically involves the knee joint, especially the medial femoral condyle; other joints involved may include the elbow, ankle, and hip [2].

OCD is more prevalent in males. The male to female ratio was approximately 2:1. In children, the most common causes that relate with the onset of OCD are activities related to sports, especially those involving repeated contact or stress to the joints. Treatment modalities also differ according to stage and range from conservative management, such as rest and physical therapy, to surgical interventions, such as microfracture or osteochondral grafting in selected cases [3].

In this report, we present an atypical case of osteochondritis dissecans occurring at a younger age than typically expected, characterized by a sudden onset of knee pain combined with a delayed initial presentation of three months. We believe this unusual presentation highlights important considerations regarding the clinical variability of OCD, the potential implications of delayed diagnosis, and the significance of an early, aggressive surgical approach. We constructed this case report using SCARE Guideline [4].

## 2 Case presentation

**Fig. 1:**
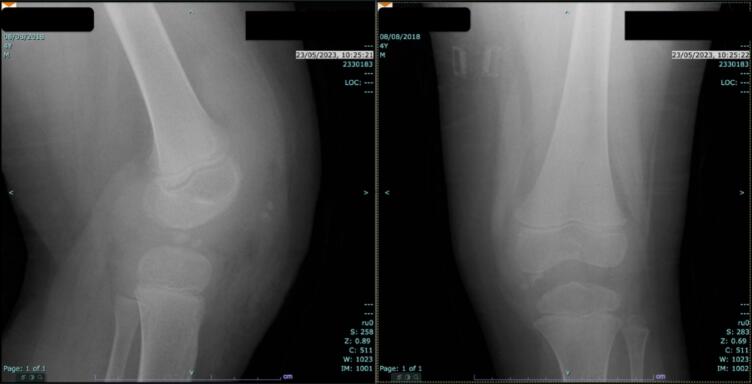
Plain radiographs of the left knee on initial presentation (Left: Lateral view; Right: Anteroposterior view).

An 8-year-old child presented with a three months of left knee pain that suddenly developed upon rising one morning. The pain mildly subsided during the day, but worsened with any knee movement. This resulted in an apparent limp over time. Clinical examination revealed that the left knee had swelling and restriction of the range of motion, including an extension deficit of 30°. The patient initially had a Tegner activity score of 2 and Lysholm knee score of 73 (Fig. 1).

Based on the radiological diagnosis, this case confirmed a 1 × 1.5 cm chondral defect on the weight-bearing area of the medial condyle surrounded by fibrous cartilage with a detached cartilage flap. Radiographic findings were consistent with those of Clanton and DeLee type III. The patient had a height of 121 cm, weight of 42 kg, with a BMI of 28.7 (Overweight).

**Fig. 2:**
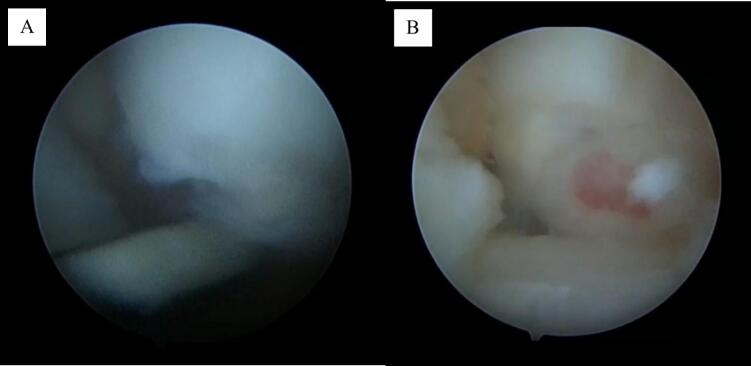
Identification of defects using arthroscope. A) Identification of OCD and B) OCD defects on the medial condyle.

The patient underwent arthroscopic surgery by means of a wrist arthroscope that allowed the precise debridement of the chondral defect and removal of the cartilage flap. The defect area was identified using an arthroscope (Fig. 2). Subsequently, microfractures were created in the chondral defect area.

**Fig. 3:**
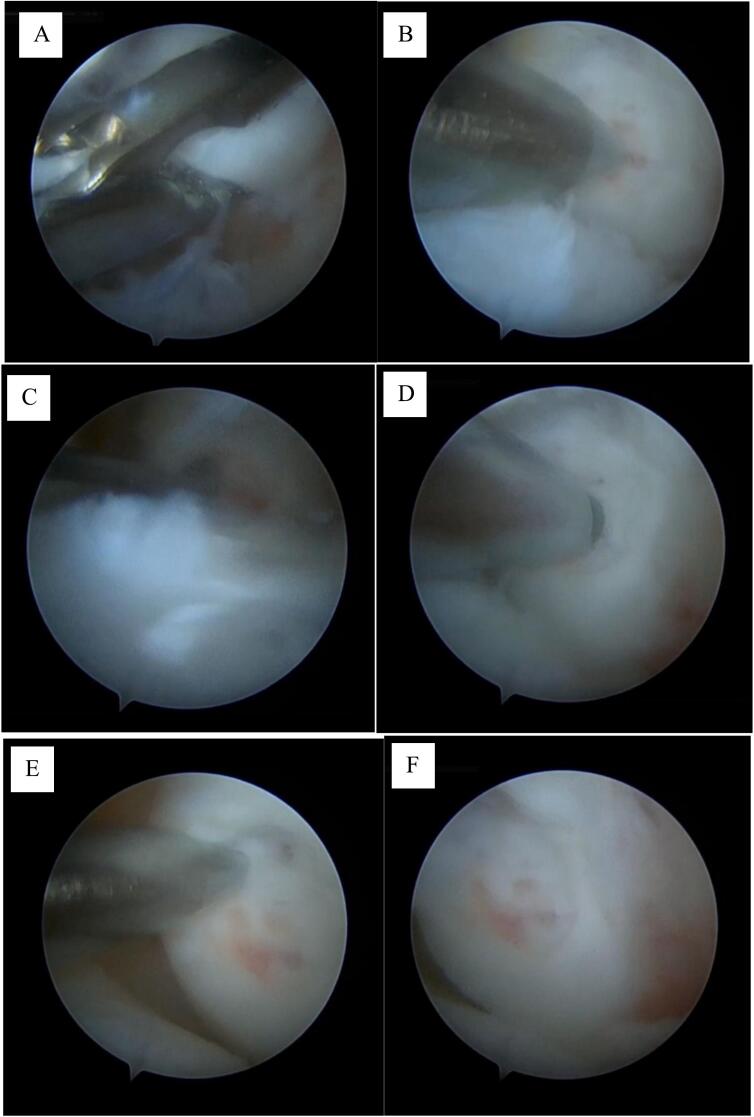
Intraoperative Procedure: A) OCD excision performed, B) microfracture performed, C) refreshing the defect on the condyle with a curette, D) defect refreshed with a Rosen burr, E) microfracture performed again, and F) evaluation of the defect after refreshing.

Fig. 3 depicts arthroscopic surgery. The procedure started with excision of the unstable osteochondral fragment, followed by debridement to eliminate loose cartilage and fibrous tissue. The exposed defects were further enlarged by the induction of microfractures. The articular cartilage defect on the condyle was debrided with a curette to eliminate the leftover debris and necrotic tissue. Subsequently, more microfracture perforations were created to improve blood circulation and augment the repair capacity. The defect margins were subsequently polished with a Rosen burr to stabilize and smoothen cartilage edges. The prepared defects were assessed by verifying a clean and adequately prepared bed conducive to fibrocartilage integration. The final result was then sealed with fibrin glue under dry arthroscopic conditions.

**Fig. 4:**
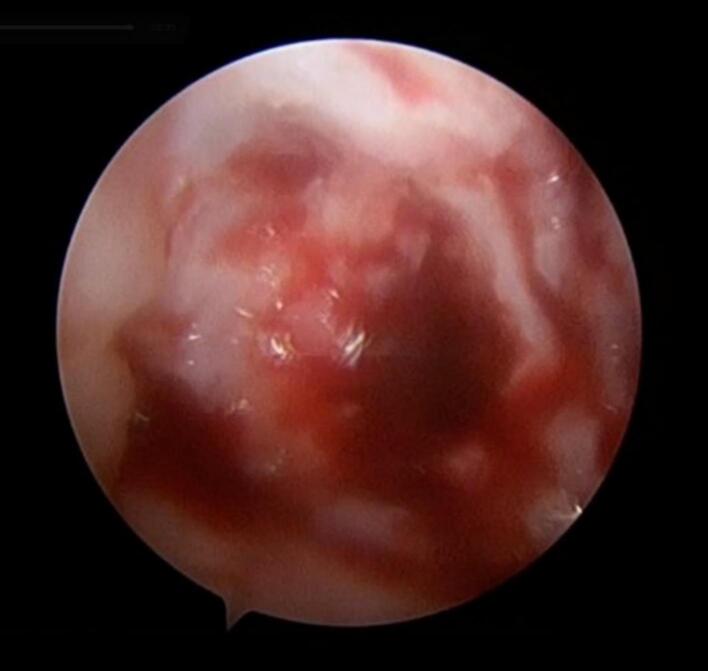
Final intraoperative image demonstrating fibrin glue application to the surgical site.

Fig. 4 depicts the osteochondral defect after the administration of fibrin glue. The adhesive conceals the imperfection, establishing a stable substrate to facilitate tissue integration and improve the healing process.

**Fig. 5:**
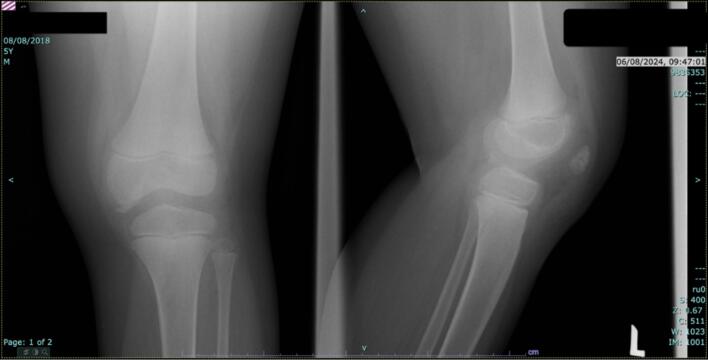
One-year postoperative plain radiographs of the left knee (AP and lateral views).

The knee was post-operatively immobilized using a Robert Jones bandage, and the patient underwent rehabilitation. The patient showed a significant postoperative improvement in pain and function. A follow-up examination 2 months later showed full knee flexion and extension to 0°, without contracture. The patient had a Lysholm knee score of 93 and Tegner activity score of 4. After one year of follow-up, the patient had no knee complaints (Fig. 5).

## 3 Clinical discussion

Treating osteochondritis dissecans (OCD) in pediatric patients presents several unique challenges owing to the smaller joint size and open growth plates typical of children. Pediatric joints, especially the knee, have smaller anatomical structures, making the use of standard adult arthroscopic instruments difficult and risky. Injuries to joint space must be taken seriously because they can lead to bone malformations due to interruption of normal bone growth [5].

Pediatric joints, especially the knee, are characterized by smaller anatomical structures, which make the use of standard adult arthroscopic instruments both difficult and risky. Injuries to the joint space can lead to bone malformations and other complications due to interrupted bone growth [6]. Advances in smaller arthroscopic devices, such as wrist arthroscopes, have shown potential for greater precision, shorter recovery times, and fewer complications [7].

Another challenge is determining the optimal treatment modality based on lesion size, location, and stability. Stability, although crucial, remains difficult to ascertain using imaging alone. Techniques such as microfracture, debridement, and scaffold-assisted arthroscopy offer tailored solutions for lesions at various stages of progression [8].

Early stage, stable lesions with preserved cartilage can often be managed conservatively. Approaches include limiting physical activity, prescribing rest, and using orthoses to promote natural healing. Evidence suggests that up to 80 % of early stage OCD lesions heal completely under conservative management, emphasizing the importance of early diagnosis [9]. For lesions that fail to respond to conservative treatment or become unstable, surgical options are typically explored. Arthroscopic techniques, such as drilling, debridement, or microfracture, aim to stimulate subchondral blood flow and enhance healing. These techniques are particularly valuable in minimizing invasiveness and protecting open growth plates in pediatric patients [10].

Emerging approaches, such as single-layer hyaluronate-based scaffolds and matrix-assisted chondrocyte implantation, have shown promise in the adult and adolescent populations. Although their application in children is still limited, they represent a growing frontier in OCD treatment [8,11]. Graft-Based Solutions: Osteochondral autograft or allograft transplantation may be used for larger or refractory lesions. These techniques have demonstrated success in restoring joint function and reducing pain [10].

A conservative approach is usually used in adolescent patients with early stage disease, especially if the lesion is stable and the cartilage is preserved. These include limiting, avoiding undue activities, resting the patient, and use of orthoses until a time when operation can be done as it is not necessary immediately [12].

When conservative treatment fails or the lesion becomes unstable owing to a loose fragment of bone and cartilage, surgical options are typically considered. By stimulating blood flow to the area, arthroscopic techniques, such as drilling or microfracture, often promote healing [5]. pediatric patients require special care to prevent damage to the growth plate. The use of smaller instruments, such as a wrist arthroscope, has shown potential to increase precision during these procedures while minimizing soft tissue damage [3]. Biodegradable pins or screws can be used to fix the lesion from the inside, and in more serious cases, osteochondral autograft or allograft transplantation can be performed.

Minimally invasive techniques are preferred in pediatric patients to reduce the risk of complications, such as postoperative stiffness, infection, or the development of early onset osteoarthritis. New techniques such as autologous chondrocyte implantation (ACI) have shown promise in adolescents and adults, but their use in children is limited due to concerns about the immature cartilage's ability to heal and assimilate properly [13].

The prognosis for pediatric patients undergoing surgical intervention is generally favorable, with success rates ranging between 70 and 90 % for return to pre-injury activity levels [6]. Factors influencing outcomes include lesion size, stage at diagnosis, and adherence to postoperative protocols. Minimally invasive arthroscopy has led to improved patient satisfaction, faster recovery times, and fewer complications than open surgery. Innovations such as autologous chondrocyte implantation and cell-free biomimetic scaffolds are paving the way for more effective treatments [14]. In this case, postoperative follow-up showed no complications, and radiographic evaluation proved that the growth plate remained open without any adverse effects on limb growth or development.

Recent systematic studies have highlighted the benefits of integrating arthroscopic microfracture procedures with bioabsorbable biomaterials, including accelerated healing, improved functional outcomes, and reduced recovery time. Basciani et al. determined that the inclusion of scaffolds or bioabsorbable materials with arthroscopic microfracture markedly improves cartilage regeneration and clinical outcomes, demonstrating distinct advantages over microfracture alone, particularly in younger patients [15]. Wiktor and Tomaszewski similarly noted enhanced healing and improved clinical outcomes when biomaterials were utilised in conjunction with arthroscopy treatment for OCD patients, thereby reinforcing the efficacy of an assertive, minimally intrusive strategy [16]. Basciani and colleagues revealed that arthroscopic microfracture, when paired with biomaterials, dramatically enhanced joint function and patient satisfaction relative to conventional methods [15]. These findings are in strong accordance with our strategy of early and assertive arthroscopic intervention utilizing fibrin glue to enhance healing, minimize recovery durations, and elevate functional outcomes, as demonstrated by our patient's swift postoperative recovery and outstanding functional ratings.

This study has several limitations that must be acknowledged. Owing to the infrequency of OCD in young pediatric patients, our centre recorded only one case, indicating an insufficient sample size. The authors acknowledge that the atypical clinical presentation in our instance, especially the younger age, rapid onset, and delayed diagnosis, may limit generalizability. This study may not have generalizability for these reasons. Further comprehensive trials or multicenter collaborations may confirm the efficacy and generalisability of this therapeutic approach in similar unusual instances, particularly in younger patients [17].

## 4 Conclusion

This case underscores the effectiveness of utilizing a wrist arthroscope for pediatric knee arthroscopy, particularly for treating OCD. The smaller instrument allowed for precise intervention, resulting in significant symptomatic relief and functional improvement. This approach addresses the gap in appropriate arthroscopic tools for pediatric joints, suggesting that smaller, more adaptable instruments could play a crucial role in optimizing outcomes in children with knee conditions. Developing and standardizing such tools may offer a feasible and effective alternative to traditional adult-sized equipment currently in use.

## CRediT authorship contribution statement

Stefanus Hengkie Marseno: study concept, data collection, manuscript preparation, manuscript editing, literature search.

Tri Wahyu Martanto: study design, manuscript review, supervision, guarantor.

Hizbillah Yazid: study design, data interpretation, manuscript review, supervision.

Arif Zulkarnain: study design, data interpretation, manuscript review, supervison.

Kukuh Dwiputra Hernugrahanto: study conept, data collection, data interpretation, manuscript review.

Julian Benedict Swannjo: literature search, data interpretation, manuscript preparation, manuscript editing.

## Informed consent

Written informed consent was obtained from the patients' parents/legal guardians for publication and for any accompanying images. A copy of the written consent is available for review by the Editor-in-Chief of this journal upon request.

## Ethical approval

The ethical approval was waived by an Institutional Review Board. Copies of informed consent are available for review by the Editor-in-Chief of the journal upon request.

## Funding

None.

## Declaration of competing interest

The authors declare no conflicts of interest.

## Appendix A Supplementary data

Supplementary video Arthoscope of pediatric osteochondritis dissecans Supplementary video
